# The Case of the Missing Dentures: A Case Report and Review of Esophageal Foreign Body as a Cause of Chest Pain

**DOI:** 10.7759/cureus.26898

**Published:** 2022-07-15

**Authors:** Kevin Pink

**Affiliations:** 1 Internal Medicine, New York Presbyterian Brooklyn Methodist Hospital, Brooklyn, USA

**Keywords:** denture, upper endoscopy, non-cardiac chest pain, chest pain, esophageal foreign body

## Abstract

Esophageal foreign body ingestion is a cause of non-cardiac chest pain and can be associated with significant mortality. Typically, esophageal foreign body ingestion is managed with endoscopic retrieval to prevent complications. The most commonly ingested foreign bodies in adults are food boluses. However, sometimes we see patients after the ingestion of more atypical and dangerous objects. Here, we present a case of a 66-year-old female who presented to the emergency department with chest pain. Quickly after admission to the emergency department, the patient was noted to have an esophageal foreign body on the chest radiograph. The patient was subsequently taken for endoscopic management of the foreign body and intubated for airway protection. With careful manipulation of the mystery object, the foreign body was removed and the patient was able to be discharged safely from the hospital without complications. This case emphasizes the importance of keeping a broad differential for one of the most commonly seen chief complaints in the emergency department to ensure timely triage of patients.

## Introduction

Chest pain is one of the most common chief complaints on presentation to the emergency department, accounting for approximately 10% of non-trauma-related cases [1]. Approximately 50% of these cases are related to acute cardiopulmonary conditions, whereas the other 50% are related to musculoskeletal, gastrointestinal, or other more obscure conditions [2-3]. Of these obscure causes, foreign body ingestion is an important differential to keep on the table. Foreign body ingestion is much more common in children than in adults, with the most commonly ingested foreign body in adults being impacted meat or food bolus [4-5]. Patients who are particularly at risk in the adult population include those with psychiatric conditions, mental disabilities, alcohol or drug abuse, and the geriatric population [6-7]. Most foreign bodies pass spontaneously without intervention. However, it has been reported that as many as 1500 deaths occur each year due to foreign body ingestion [6-7]. Complications of foreign body ingestion include esophageal perforation, mediastinitis, and fistula formation [8]. Timely diagnosis improves outcomes and prevents complications. Here, we present a case of esophageal foreign body, presenting as chest pain, to analyze how providers can more efficiently diagnose and treat this condition.

## Case presentation

**Figure 1:**
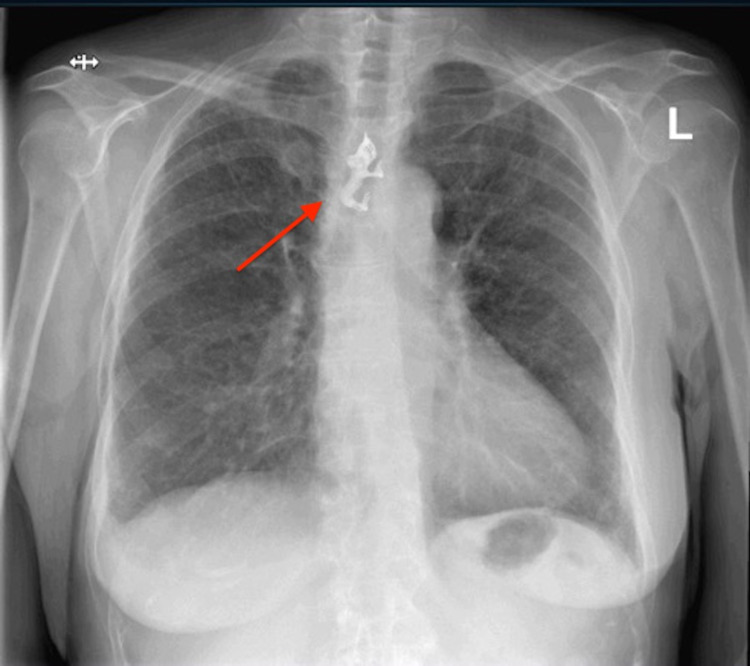
A chest radiograph demonstrating a radiopaque metallic dental bridge which is swallowed and trapped in the proximal third of the thoracic esophagus. Arrow pointed at the foreign body.

A 66-year-old woman with a history of epilepsy, which is currently well controlled with levetiracetam, presented to the emergency room with chest pain. The patient stated that the chest pain was substernal and sharp in character without radiation. The patient stated that the pain started in the morning and had been constant since the onset. All the other remaining reviews of systems were negative. The patient did not endorse any personal or family history of cardiopulmonary disease. She also denied smoking, drinking, and recreational drug use. An initial physical exam showed normal vital signs, a patient in mild painful distress, and an otherwise benign physical exam. Initial laboratory studies were largely within normal limits. An electrocardiogram revealed normal sinus rhythm with a ventricular rate of 78 beats per minute. A chest radiograph revealed a radiopaque metallic dental bridge, swallowed and trapped in the proximal third of the thoracic esophagus (Figure 1).

**Figure 2:**
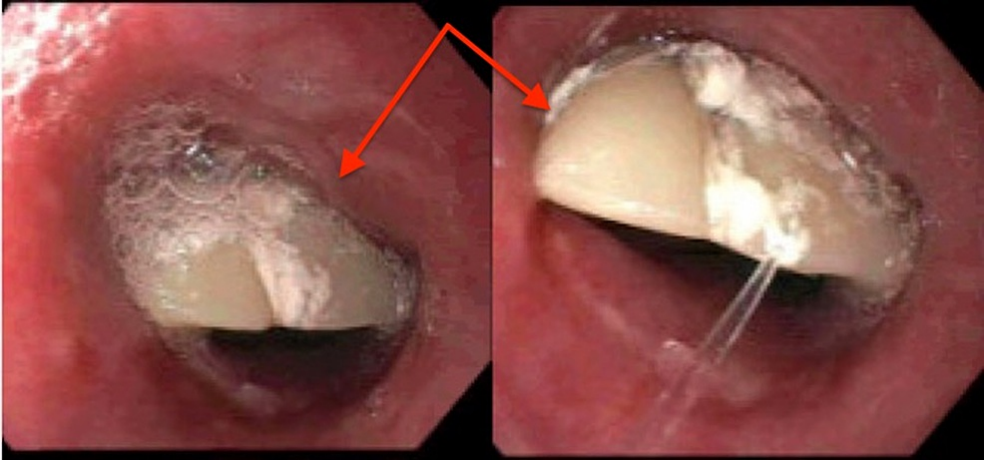
Partial denture noted in the upper (left) and middle (right) portions of the esophagus. Arrows pointed at the denture.

**Figure 3:**
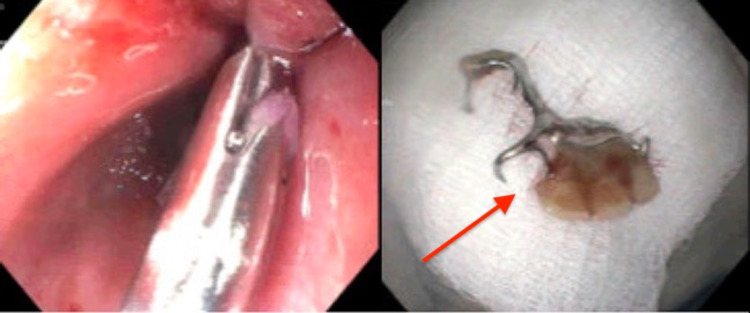
The left image demonstrates the area of the esophagus where the denture was previously sitting and the right image showed the denture itself after successful removal. Arrow pointed at denture after removal.

Further history revealed that the patient had fallen asleep the night prior with her dentures in place and when she woke up in the morning she noticed they had gone missing. The patient stated that she had not attempt to eat or drink anything and was noted at this time to be tolerating secretions well. The patient was quickly taken to the operating room for emergent endoscopic procedure with the gastrointestinal team at which time she was intubated for airway protection. She was evaluated with a flexible endoscope. Initial attempts to remove the foreign body with a large loop snare were unsuccessful and the procedure was aborted for evaluation by cardiothoracic surgery and the advanced endoscopy team. On repeat endoscopic evaluation the foreign body was once again noted in the esophagus. At this time, the denture was able to be carefully advanced into the stomach and was retrieved with rat tooth forceps. The endoscope was then reinserted into the esophagus to evaluate the success of the procedure, at that time, a small superficial mucosal tear was noted and three hemostatic clips were placed (Figures 2-3).

The patient tolerated the procedure well and was subsequently monitored in an intensive care unit setting. The patient was initially kept nil per os with a plan to slowly advance the diet. A fluoroscopic esophagram was completed on postoperative day 1, which showed no radiographic evidence of a foreign body and new endoclips without tear, stricture, or mucosal irregularity. The patient did not experience any complications from the procedure and was able to be discharged home the following day. 

## Discussion

With chest pain being one of the most common presenting symptoms to the emergency department, it is important that esophageal foreign bodies be kept in the differential. Adult foreign body ingestion carries significant morbidity and mortality [8].

During the initial evaluation of chest pain, life-threatening conditions such as acute coronary syndrome, acute aortic dissection, pulmonary embolism, tension pneumothorax, pericardial tamponade, mediastinitis, and esophageal foreign bodies should be evaluated for. History and physical examination can be useful in assigning likelihood ratios to each of the aforementioned diagnoses [9]. These predictive values can be helpful in deterring whether the next steps include an electrocardiograph, chest radiograph, cardiac enzymes, or in this case, an evaluation by a gastroenterologist.

On the initial evaluation of patients with foreign body ingestion, it is important to evaluate for drooling and the ability to tolerate secretions as this can incur a significant aspiration risk. For patients not tolerating secretions, it is important to ensure timely airway management, as well as urgent endoscopic or surgical intervention [9]. Current guidelines recommend that imaging modalities including computed tomography and radiography be performed in patients not requiring emergent intervention [9] Indications for emergent intervention include esophageal obstruction and the presence of disk-shaped batteries or sharp objects (dentures, fish bones, toothpicks) [10-12].

Forward-viewing flexible endoscopes are the instruments of choice due to their enhanced ability to retrieve foreign objects and evaluate affected mucosa [10]. Both flexible and rigid endoscopes have high success rates; however, rigid endoscopes are associated with a higher risk of perforation [13-14]. Rat-tooth forceps and the snare are frequently used retrieval devices, but other retrieval devices include alligator forceps, nets, and baskets. Following successful endoscopic treatment, most patients are able to be managed as outpatients, whereas patients with unsuccessful endoscopic treatment are typically hospitalized and monitored with serial radiographic exams and follow-up endoscopy. Indications for inpatient observation include difficult extraction and ingestion of multiple objects or objects associated with perforation. Surgical intervention is indicated in patients who develop perforation or have high-risk objects unable to be extracted by endoscopy [15].

This patient presented with chest pain and was subsequently found to have an esophageal foreign body. At the time of the initial history, it is likely the provider had anchored on more common causes of chest pain such as acute coronary syndrome, pulmonary embolism, or gastro-esophageal reflux disease. A more careful history could have allowed the provider to surpass the unnecessary step of obtaining chest radiography and allow the patient to be more appropriately triaged. Fortunately, this patient was able to be safely managed after the diagnosis was made without procedural complications. An important identifiable risk factor in the patient may have been epilepsy.

## Conclusions

Chest pain is an extremely common complaint on presentation to the emergency department and this differential diagnosis can vary widely. It is important that physicians use careful history-taking and physical examination to better guide subsequent diagnostic testing and shorten the time to contact appropriate consultation services. Esophageal foreign body ingestion, although more common in children, can occur in the adult population and should always be considered in patients complaining of chest pain. Deadly complications such as perforation, mediastinitis, and fistula formation can be prevented with timely diagnosis and early endoscopic evaluation.
